# Accompaniment to healthcare visits: the impact of sensory impairment

**DOI:** 10.1186/s12913-020-05829-8

**Published:** 2020-10-29

**Authors:** Nicholas S. Reed, Lama Assi, Emily Pedersen, Yasmeen Alshabasy, Ashley Deemer, Jennifer A. Deal, Amber Willink, Bonnielin K. Swenor

**Affiliations:** 1 Cochlear Center for Hearing and Public Health, Johns Hopkins University Bloomberg School of Public Health, 2024 E. Monument Street, Baltimore, MD 21205 USA; 2 Department of Epidemiology, Johns Hopkins University Bloomberg School of Public Health, Baltimore, MD USA; 3 Wilmer Eye Institute, Johns Hopkins University School of Medicine, Baltimore, MD USA; 4 Department of Health Policy and Management, Johns Hopkins University Bloomberg School of Public Health, Baltimore, MD USA

**Keywords:** Hearing, Vision, Dual sensory impairment, Accompaniment, Companion

## Abstract

**Background:**

Millions of older adults in the United States experience hearing, vision, and dual sensory impairment (concurring hearing and vision impairment) yet little research exists on their needs in interactions with the healthcare system. This piece aims to determine the use of accompaniment in healthcare interactions by persons with sensory impairment.

**Methods:**

These cross-sectional analyses included data from the 2015 Medicare Current Beneficiaries Survey and survey weighting provided by Centers for Medicare and Medicaid Services. Adjusted odds of reporting accompaniment to healthcare visits and given reasons for accompaniment among United States Medicare beneficiaries with self-reported sensory impairment (hearing, vision, and dual sensory impairment) were examined.

**Results:**

After excluding observations with missing data, 10,748 Medicare beneficiaries remained representing a 46 million total weighted nationally representative sample, of which 88.9% reported no sensory impairment, 5.52% reported hearing impairment, 3.56% reported vision impairment, and 0.93% reported dual sensory impairment. Those with vision impairment and dual sensory impairment had 2.139 (95% confidence interval [CI] =1.605–2.850) and 2.703 (CI = 1.549–4.718) times the odds of reporting accompaniment to healthcare visits relative to those without sensory impairment. A secondary analysis suggests communication needs as the primary reason for accompaniment among persons with hearing loss, while those with vision impairment were more likely to indicate transportation needs.

**Conclusions:**

Healthcare accompaniment is common for persons with sensory loss and healthcare systems should consider accommodations for and leveraging accompaniment to improve healthcare for persons with sensory impairments. In light of the current COVID-19 pandemic, as hospitals limit visitors to reduce the spread of infection, arrangements should be made to ensure that the communication and transportation needs of those with sensory impairment are not neglected.

## Background

### Sensory impairment

Vision and hearing impairment are highly prevalent, chronic conditions which disproportionately affect older adults [1]. In the United States, vision impairment impacts approximately 14 million individuals 12 years and older, of which approximately 3 million would remain impaired even with refractive correction [2]. Similarly, estimates suggest that hearing impairment, defined as a mild or greater hearing loss using World Health Organization criteria, impacts 38 million Americans and increases in prevalence with age such that two-thirds of Americans over age 70 years [3, 4]. A small and often overlooked subset is those with dual sensory impairment, concurrent hearing and vision impairment, which affects 1 in 9, or 11.3%, of Americans 80 years or older [5]. As the United States population ages, these numbers are expected to increase [6, 7].

Once considered benign aspects of aging, recent literature has emphasized the public health importance of sensory impairment. Sensory impairment is independently associated with numerous negative health outcomes in older adults, including cognitive decline, dementia, falls, and functional decline and loneliness [8–13]. Despite consequences outlined above, adoption and pursuit of sensory care remains low: only 20–30% of persons with hearing impairment own and use hearing aids while less than 50% of Medicare beneficiaries with vision impairment have had an annual eye exam [14, 15].

Concurrent vision and hearing impairment may exacerbate negative health associations. Dual sensory impairment presents a unique challenge by removing sensory substitution compensatory strategies (i.e. reliance upon visual cues in the presence of hearing impairment and auditory cues in the presence of visual impairment) [16]. There is a paucity of research on adults with dual sensory impairment. However, available evidence suggests those with dual sensory impairment experience compounded psychosocial, psychological and functional effects compared to those with either hearing or visual impairment only [17].

### Sensory impairment in healthcare

Recent research has focused on how sensory impairment impacts healthcare specific outcomes. In a study of matched adults with and without vision loss who were hospitalized for common illnesses, Medicare beneficiaries with severe vision loss had, on average, 4% longer lengths of stay, 22% higher odds of 1-month readmission, and 12% higher mean costs of healthcare services during hospitalization and 90 days after discharge. Similar associations were seen among adults with commercial health insurance, where those with severe vision loss had a 4% longer length of stay, 32% higher odds of readmission, and 8% higher costs [18]. Similarly, in a study of propensity-matched adults with and without hearing loss, adults with hearing loss accumulated, on average, $22,434 (95% CI, $18,219–$26,648) more in healthcare expenditures over a 10-year period. In addition, adults with hearing loss experienced a 47% higher rate of hospitalization, 44% higher risk of 30-day readmission, and spend, on average, 2.5 days longer during an inpatient stay over a 10-year period [19]. Increased healthcare utilization may contribute to suggested poorer perceived satisfaction with care among those with sensory loss [20, 21].

Sensory impairment may negatively impact and limit communication, including patient-provider communication. When navigating health systems, individuals with vision or hearing impairment face barriers to effective communication which is a pillar of patient-centered care [22]. For clinicians, communication limitations with patients could increase the time, effort and frustration associated with providing adequate care. For those with sensory impairment, inadequate communication with healthcare providers could increase the likelihood of misunderstanding medical information, reduce treatment adherence, lower patient satisfaction, and can result in poorer clinical outcomes or even accidental injury [23].

### Accompaniment in healthcare

In the United States, 20–50% of older adult patients are accompanied by a companion to their medical visits [24]. Importantly, in response to deficits in care quality and rising healthcare costs, there is a growing appreciation for patient-centered care and patient-provider communication and the concept of “patient-family-physician” partnerships. Previous studies on accompaniment in healthcare settings have demonstrated that the presence of companions may positively impact patient understanding, information recall and engagement in medical decision-making [25, 26]. However, there is limited research on the patterns of accompaniment for older adults with sensory impairment despite their unique communication and physical (i.e., transportation) barriers faced in accessing and interacting with the healthcare system. For older adult patients with sensory impairment, accompaniment may be particularly important to optimal patient care. To our knowledge, no previous studies have examined whether sensory impairment is associated accompaniment to physician visits in a nationally representative sample of older adults. This study investigated the patterns of and reasons for accompaniment to physician visits of Medicare beneficiaries with hearing impairment, vision impairment, and dual sensory impairment compared to those without sensory impairment.

## Methods

### Data source

This cross-sectional, retrospective cohort study was conducted using de-identified data from the Medicare Current Beneficiary Survey (MCBS) 2015 public use file [27]. The MCBS is an annual nationally-representative survey of Medicare beneficiaries which collects demographic, socioeconomic, medical and health status, healthcare utilization, healthcare access, and healthcare satisfaction data. Medicare is the United States federal health insurance program for all adults aged 65 and older as well as adults younger than 65 with disabilities and end stage renal disease [28]. In 2015, a reported 55,496,222 individuals were enrolled in Medicare [29]. Of these enrollees, 12,311 Medicare beneficiaries participated in the 2015 MCBS. Due to the public availability and de-identified nature of the data set, this study was exempt from institutional review board.

### Outcome variables

The primary and secondary outcomes of this study were accompaniment to the doctor's office and reasons for accompaniment, respectively. Participants were first asked if they had a usual place of care when sick or in need of medical advice. Those who noted a usual place of care were asked: “Do you usually have someone accompany you there [usual place of care]?” – “No” or “Yes”. In follow-up, participants were asked “What are the reasons this person accompanies you there? What does this person do?” They were then given a list of responses and asked to check all that apply. The responses included: “writes down what the doctor says/records instructions/takes notes/remembers,” “gives information/explains medical conditions or needs to doctor,” “explains doctor’s instructions to [participant],” “asks questions,” “translates language,” “schedules appointments,” “nothing/keeps company/sits with [participant]/moral support,” “transportation,” “[participant] needs physical assistance, and “other.”

### Exposure and covariates

Medicare beneficiaries were categorized into sensory impairment categories based on self-report questions. Participants were asked, “Which statement best describes your hearing [with a hearing aid]?” - “No trouble”, “A little trouble”, and “A lot of trouble” and “Which statement best describes your hearing [with a hearing aid]?” - “No trouble”, “A little trouble”, and “A lot of trouble” and “Which statement best describes your vision [while wearing glasses]?” - “No trouble”, “A little trouble”, and “A lot of trouble.” Participants were than categorized according to responses to these questions: (1) no perceived sensory impairment, (2) perceived hearing impairment only, (3) perceived vision impairment only, and (4) perceived dual sensory impairment. Participants were classified as having perceived dual sensory impairment if they replied “a lot of trouble” to both questions. They were classified as having perceived hearing impairment only if they replied “a lot of trouble” to only the hearing question and classified as having perceived vision impairment only if they replied “a lot of trouble” to only the vision question. All other participants were classified as having no perceived sensory impairment. Limiting to “a lot of trouble” was selected to capture participants who are significantly impacted and impaired by sensory impairment.

Several factors were identified as potential confounders. Demographic variables included age (categorized as younger than 65, 65 to 74 years old, and older than 75 years), self-reported race (Non-Hispanic White, Non-Hispanic Black, Hispanic, and other), and gender (male or female). Further, we included sociodemographic variables including educational attainment (less than high school, high school or vocational/technical/business degree, more than high school), income (less than $25,000 or greater than or equal to $25,000), dual Medicaid-Medicare eligibility status (nondual, full dual, partial dual, and qualified Medicare beneficiary dual), and marital status (married or not married/widowed/divorced). Health-related factors included self-perceived general health status compared to others of the same age (excellent, very good, good, fair, or poor), self-reported depression based on ever being told by a doctor (yes or no), and difficulty walking ¼ mile (no difficulty, little difficulty, some difficulty, a lot of difficulty, or unable to do it).

### Statistical analysis

Survey weights, supplied by Centers for Medicare and Medicaid Services, were applied to account for the design of the MCBS including stratification, clustering, multiple stages of selection, and disproportionate sampling. Descriptive and univariate chi-square analyses were used to identify trends and explore associations between sensory impairment categories, covariates, and outcome variables. Multivariate logistic regression was used to model the association between perceived sensory impairment and odds of accompaniment after adjusting for confounding variables. A model building approach was utilized using Akaike Information Criterion to assess models. The β-coefficients (log-odds) were converted into odds ratios for ease of interpretation. Significance testing for all analyses was 2-sided with a type I error of 0.05. Subjects with missing data were excluded from analyses. Sensitivity analysis excluding those with a Medicare status code indicating disabled (i.e., receiving Medicare for reasons other than age) was completed. The statistical software used was Stata 15 (StataCorp, College Station, TX).

## Results

### Demographic characteristics

Following exclusion of those with missing data, the final unweighted sample included 10,748 Medicare Beneficiaries. In a nationally representative weighted sample of 46,029,364 Medicare beneficiaries in 2015, 41,421,015 (88.9%) reported no sensory impairment, 2,542,752.2 (5.52%) reported hearing impairment only, 1,637,622.5 (3.56%) reported vision impairment only, and 427,974.7 (0.93%) reported dual sensory impairment. Table 1 displays the weighted demographic characteristics of this sample. Among Medicare beneficiaries without sensory impairment, 32.59% reported having someone accompany them to physician visits in 2015, compared to 65.52% of those with vision impairment, 43.46% of those with hearing impairment, and 72.11% of those with dual sensory impairment. A higher proportion of Medicare beneficiaries with dual sensory impairment (59.85%) and hearing impairment (45.23%) were 75 years and older than those with vision impairment (35.96%) and no sensory impairment (33.33%). A higher proportion of those with vision impairment identified as Non-Hispanic Black (13.72%) or Hispanic (15.38%) relative to those with no sensory impairment, hearing impairment, and dual sensory impairment. Relative to individuals without sensory impairment, Medicare beneficiaries with sensory impairment (hearing, vision, or dual-sensory impairment) reported a lower proportion of individuals with more than a high school diploma, a higher proportion of persons reporting income of less than $25,000 a year, and lower proportions of married individuals. A higher proportion of participants with dual sensory impairment reported inability to walk a quarter mile (43.70%) and poor health relative to others their age (19.31%) compared to those with hearing impairment (26.39, 12.16%), vision impairment (36.12, 23.98%), and those with no sensory impairment (13.16, 5.97%).

**Table 1:** Demographic and Socioeconomic Characteristics of Medicare Beneficiaries by Self-Reported Sensory Impairment

		Self-Reported Sensory Impairment		
	No Sensory Impairment	Hearing Impairment	Vision Impairment	Dual Sensory Impairment
Variable	N (%)	N (%)	N (%)	N (%)
**Unadjusted sample**				
(*N* = 10,748)	9524 (88.61%)	668 (6.22%)	435 (4.05%)	121 (1.13%)
**Weighted sample (In Millions)**				
(*N* = 46.03)	41.42 (89.99%)	2.54 (5.52%)	1.64 (3.56%)	0.43 (0.93%)
**Age (years)**				
64 and younger	6.20 (14.98%)	0.39 (15.27%)	0.49 (29.88%)	0.081 (18.98%)
65–74	21.41 (51.69%)	1.00 (39.5%)	0.56 (34.16%)	0.09 (21.17%)
75 and older	13.80 (33.33%)	1.15 (45.23%)	0.59 (35.96%)	0.26 (59.85%)
**Gender**				
Female	23.11 (55.79%)	1.13 (44.4%)	1.02 (62.28%)	0.23 (53.72%)
**Race**				
Non-Hispanic White	31.28 (75.51%)	2.05 (80.48%)	1.00 (61.1%)	0.31 (71.69%)
Non-Hispanic Black	3.89 (9.38%)	0.13 (4.98%)	0.22 (13.72%)	0.02 (4.94%)
Hispanic	3.47 (8.38%)	0.17 (6.78%)	0.25 (15.38%)	0.04 (9.68%)
Other	2.79 (6.73%)	0.20 (7.77%)	0.16 (9.79%)	0.06 (13.68%)
**Educational attainment**				
Less than 9th grade	6.16 (14.87%)	0.60 (23.64%)	0.45 (27.72%)	0.17 (39.03%)
High school or vocational degree	14.42 (34.82%)	1.00 (39.37%)	0.72 (43.46%)	0.15 (34.04%)
More than high school	20.84 (50.30%)	0.94 (36.99%)	0.47 (28.82%)	0.12 (26.93%)
**Income**				
Less than $25,000	15.22 (36.74%)	1.10 (43.35%)	0.96 (58.52%)	0.30 (71.06%)
**Marital Status**				
Married	23.07 (55.71%)	1.35 (53.02%)	0.72 (43.99%)	0.13 (30.40%)
**Difficulty walking ¼ mile**				
No difficulty	23.75 (57.33%)	0.86 (33.67%)	0.39 (24.09%)	0.06 (13.80%)
Little difficulty	4.48 (10.82%)	0.23 (8.97%)	0.13 (8.05%)	0.03 (7.72%)
Some difficulty	4.32 (10.43%)	0.41 (16.21%)	0.27 (16.23%)	0.08 (19.10%)
A lot of difficulty	3.42 (8.26%)	0.38 (14.77%)	0.25 (15.51%)	0.07 (15.67%)
Unable to do it	5.45 (13.16%)	0.67 (26.39%)	0.59 (36.12%)	0.19 (43.70%)
**General health**				
Excellent	7.46 (18%)	0.20 (7.93%)	0.10 (6.01%)	0.02 (5.66%)
Very good	12.61 (30.45%)	0.56 (22.18%)	0.24 (14.49%)	0.04 (10.34%)
Good	1.23 (29.66%)	0.81 (31.74%)	0.38 (22.95%)	0.13 (31.47%)
Fair	6.59 (15.91%)	0.66 (26%)	0.53 (32.57%)	0.14 (33.22%)
Poor	2.47 (5.97%)	0.31 (12.16%)	0.39 (23.98%)	0.08 (19.31%)
**Medicare Medicaid dual eligibility**				
Non dual	34.79 (83.99%)	2.04 (80.38%)	1.05 (64.02%)	0.29 (67.42%)
Full dual	4.33 (10.45%)	0.29 (11.44%)	0.44 (26.57%)	0.09 (21.12%)
Partial dual	1.31 (3.15%)	0.13 (5.29%)	0.06 (3.82%)	0.02 (5.76%)
QMB dual	1.00 (2.41%)	0.07 (2.89%)	0.09 (5.58%)	0.02 (5.70%)
**Depression**				
Diagnosed	11.55 (27.89%)	0.89 (34.98%)	0.79 (48.07%)	0.21 (48.56%)
**Does someone accompany to doc office**				
Yes	13.50 (32.59%)	1.11 (43.46%)	1.07 (65.52%)	0.31 (72.11%)

### Odds of accompaniment

Table 2 displays the odds of accompaniment by demographic and health characteristics of the weighted sample. Relative to Medicare Beneficiaries without sensory impairment, those with hearing impairment had similar odds of accompaniment (odds ratio [OR] = 1.043; 95% confidence interval [CI] = 0.841–1.293; *p* = 0.698). However, those with vision impairment (OR = 2.139; CI = 1.605–2.850; *p* = 0.000) and dual sensory impairment (OR = 2.703; CI = 1.549–4.718; *p* = 0.001) had more than 2-times the odds of reporting accompaniment than those without sensory impairments. Compared with Medicare beneficiaries 64 years and younger, those 65–74 years of age had half the odds to be accompanied (OR = 0.544; CI = 0.440–0.673; *p* = 0.000), while those 75 years and older were more likely to report accompaniment (OR = 1.382; CI = 1.163–1.643; *p* = 0.000). Compared to non-Hispanic White Medicare beneficiaries, non-Hispanic Black beneficiaries (OR = 1.221; CI = 0.999–1.492; *p* = 0.051), Hispanic beneficiaries (OR = 1.65; CI = 1.376–1.979; p = 0.000) and beneficiaries who reported ‘other’ for race (OR = 1.410; CI = 1.113–1.786; *p* = 0.005) had greater odds of accompaniment. No difference in odds was found between male and female beneficiaries. Higher income and more education were associated with reduced odds of accompaniment. Married Medicare beneficiaries had higher odds of accompaniment (OR = 2.789; CI = 2.465–3.156; *p* = 0.000) relative to those who were not married. Physical ability appeared to be strongly associated with odds of accompaniment. Indeed, compared to Medicare beneficiaries who reported no difficulty walking one-quarter mile, those who reported a lot of difficulty walking one-quarter mile (OR = 2.360; CI = 1.887–2.951; *p* = 0.000) or being unable to do this task (OR = 3.524; CI = 2.949–4.212; p = 0.000) reported higher odds of accompaniment. Similarly, those who reported poor health had higher odds of accompaniment relative to those in excellent self-reported health.

**Table 2:** Odds of accompaniment of Medicare Beneficiaries by Self-Reported Sensory Impairment

Variable	Odds Ratio (95% Confidence Interval)	Standard Error	***P***-Value
**Sensory impairment**			
No sensory impairment	REF		
Hearing impairment only	1.043 (0.841–1.293)	0.113	0.698
Vision impairment only	2.139 (1.605–2.850)	0.310	0.000
Dual sensory impairment	2.703 (1.549–4.718)	0. 759	0.001
**Age (years)**			
64 and younger	REF		
65–74	0.544 (0.440–0.673)	0.058	0.000
75 and older	1.382 (1.163–1.643)	0.120	0.000
**Gender**			
Male	REF		
Female	1.063 (0.942–1.200)	0.065	0.316
**Race**			
Non-Hispanic White	REF		
Non-Hispanic Black	1.220 (0.999–1.492)	0.123	0.051
Hispanic	1.650 (1.376–1.979)	0.151	0.000
Other	1.410 (1.113–1.786)	0.168	0.005
**Educational attainment**			
Less than 9th grade	REF		
High school or vocational, degree	0.763 (0.643–0.907)	0.066	0.002
More than high school	0.499 (0.420–0.594)	0.044	0.000
**Income**			
Less than $25,000	REF		
Greater or equal to $25,000	0.758 (0.652–0.883)	0.058	0.000
**Marital Status**			
Not married	REF		
Married	2.789 (2.465–3.16)	0.174	0.000
**Difficulty walking ¼ mile**			
No difficulty	REF		
Little difficulty	1.449 (1.190–1.765)	0.144	0.000
Some difficulty	1.364 (1.135–1.639)	0.126	0.001
A lot of difficulty	2.360 (1.887–2.951)	0.266	0.000
Unable to do it	3.524 (2.949–4.212)	0.317	0.000
**General health**			
Excellent	REF		
Very good	1.303 (1.095–1.550)	0.114	0.003
Good	1.647 (1.388–1.954)	0.142	0.000
Fair	2.116 (1.722–2.601)	0.220	0.000
Poor	2.672 (1.941–3.678)	0.430	0.000
**Medicare Medicaid dual eligibility**			
Non-dual	REF		
Full dual	1.722 (1.388–2.135)	0.187	0.000
Partial dual	1.351 (1.004–1.816)	0.202	0.047
QMB dual	1.271 (0.889–1.817)	0.229	0.185
**Depression**			
Never been diagnosed	REF		
Diagnosed	0.945 (0.831–1.075)	0.061	0.389

### Reasons for accompaniment

Table 3 displays the reasons for accompaniment among Medicare beneficiaries who indicated they were accompanied to healthcare visits by sensory impairment. In general, within each reason for accompaniment a higher percentage of persons with dual sensory impairment identified positively with the reason relative to those with no sensory impairment and/or those with hearing or vision impairment (note: participants could identify multiple reasons for accompaniment). When comparing those with hearing loss and those with vision loss relative to one another, a higher percentage of Medicare beneficiaries with hearing impairment indicated communication-related reasons for accompaniment, while a higher percentage of those with vision impairment indicated physical-related reasons for accompaniment. Indeed, 55.38% of beneficiaries with hearing impairment reported someone accompanying them to take notes compared with only 38.2% of beneficiaries with vision impairment. Similar trends were seen when the reason was explaining things to the doctor (53.77% vs. 42.93%), explaining instructions (47.26% vs. 36.76%), and asking questions (55.34% vs. 40.29%). However, Medicare beneficiaries with hearing impairment were less likely to report transportation as a reason for accompaniment than those with vision impairment, 60.51% compared to 80.47% respectively. Additionally, a lower percentage of Medicare beneficiaries with hearing impairment reported assistance with ADLs as a reason for accompaniment (19.41% vs. 28.34%). Notably, accompaniment for translation was similar regardless of sensory loss status or type.

**Table 3:** Reason indicated for Accompaniment Among Medicare Beneficiaries who reported Accompaniment by Self-Reported Sensory Impairment

		Self-Reported Sensory Impairment		
	No Sensory Impairment	Hearing Impairment	Vision Impairment	Dual sensory Impairment
Variable	N (In Millions) (%)	N (In Millions) (%)	N (In Millions) (%)	N (In Millions) (%)
**Someone accompanies to take notes**	5.50 (40.73%)	0.61 (55.38%)	0.41 (38.2%)	0.18 (58.5%)
**Someone accompanies to explain things to doctor**	5.06 (37.53%)	0.59 (53.77%)	0.46 (42.93%)	0.20 (64.93%)
**Someone accompanies to explain instructions**	4.04 (29.94%)	0.52 (47.26%)	0.39 (36.76%)	0.22 (57.6%)
**Someone accompanies to ask questions**	5.40 (40%)	0.61 (55.34%)	0.43 (40.29%)	0.20 (65.39%)
**Someone accompanies to translate**	0.48 (3.5%)	0.04 (3.22%)	0.03 (3.25%)	0.01 (3.18%)
**Someone accompanies to schedule appointment**	3.07 (22.78%)	0.33 (29.5%)	0.30 (28.07%)	0.13 (43.73%)
**Someone accompanies for moral support**	5.32 (39.41%)	0.35 (31.68%)	0.31 (29.23%)	0.06 (18.82%)
**Someone accompanies for transportation**	7.59 (56.27%)	0.67 (60.51%)	0.86 (80.47%)	0.25 (82.14%)
**Someone accompanies to assist with ADLs**	1.89 (14%)	0.21 (19.41%)	0.30 (28.34%)	0.10 (32.67%)
**Someone accompanies for another reason**	0.77 (5.68%)	0.06 (5.44%)	0.02 (1.42%)	0.03 (5.43%)

## Discussion

Our study demonstrates that in a model adjusted for demographic and health factors, Medicare beneficiaries with vision impairment and dual sensory impairment have relatively high odds of reporting being accompanied by a companion to healthcare visits relative to without sensory impairment, even after accounting for potential confounders. When examining the reasons indicated for accompaniment by sensory impairment status and type, a higher percentage of those with hearing impairment report communication-related reasons for accompaniment relative to those with vision impairment. Our study is limited by MCBS survey questions, and its respondents’ interpretation, memory, veracity and accuracy. The interpretation of MCBS survey questions about accompaniment may have included companions who remained outside of examination rooms. Thus, our findings do not account for the presence or nature of the accompaniment during the patient-physician interaction specifically, but instead more broadly describe accompaniment to physician visits. These results have implications for healthcare quality planning and adaption to accommodate for the elevated accompaniment needs of Medicare beneficiaries with sensory impairment.

To our knowledge this is the first analysis of accompaniment to physician visits by sensory impairment in a nationally representative sample. A large proportion of Medicare beneficiaries with sensory loss report accompaniment to healthcare visits relative to those without sensory loss. Much of the previous work has focused on and highlighted the beneficial aspects of accompaniment to healthcare visits including patient engagement and healthcare outcomes [25, 26, 30]. Notably, among demographic covariates, our results are consistent with previous studies which have described older adults who report accompaniment to physician visits as older, less educated by degree attainment, and poorer in health [25, 26]. The relatively high rate of accompaniment among those who identify as Hispanic may be due to language barriers [31]. Another first-in-kind finding is the difference in the reasons indicated for accompaniment, especially between Medicare beneficiaries with hearing impairment only versus vision impairment only. A higher proportion of those with hearing impairment reported communication-related reasons while a higher proportion of those with vision impairment reported physical barriers such as transportation or ADLs as reason for accompaniment. These results are consistent with previous studies indicating hearing impairment’s limitations on communication and vision impairment’s association with reduced mobility [32, 33], and access to care barriers due to transportation issues [34].

By focusing on the association with healthcare accompaniment and reasons for accompaniment among those with sensory loss, this study highlights an often-overlooked population. In particular, this piece contributes to the literature by representing persons with dual sensory impairment as vision and hearing are sometimes treated and discussed as independent of one another despite significant overlap among older adults [5]. While accompaniment is considered beneficial, the relatively high odds among persons with hearing, vision, and dual sensory impairment may indicate that the healthcare system does not appropriately accommodate persons with sensory impairment. Communication is fundamental to patient-centered healthcare [35] and ability to access healthcare relies on ability to interact with environments. As the availability of primary family resources and informal caregivers is expected to decrease with time [36], more older adults might find themselves without a companion. Healthcare systems could take steps such as implementing communication training programs specific to sensory deficits [37] and taking vision impairment into consideration when designing healthcare environments (e.g., large print signage, auditory cues).

In addition, companions are a potentially valuable quality of care resource for persons with sensory impairment [26]. Previous studies on accompaniment during patient-physician encounters suggest that companions may facilitate physician and patient understanding when they are more verbally active and engage in autonomy-enhancing behaviors [25, 26, 30, 38]. These helpful companion behaviors include recording physician instructions, providing information on the patient’s condition, asking for clarification, and offering further explanation when confusion arises. However, companions have also been shown to engage in disruptive behaviors, like interrupting or criticizing the patient, which deter patient-participation in medical decision-making [39]. Given that companionship among persons with sensory loss may be particularly important to ensure high-quality patient-provider communication and the high prevalence of accompaniment among persons with sensory loss, healthcare systems could focus on quality initiatives aimed at educating companions about the needs of persons with sensory lost to help facilitate the exchange of information.

In the context of the ongoing coronavirus disease 2019 (COVID-19) pandemic, when banning or limiting visitors at healthcare facilities may be necessary to control the spread of infection [40], the healthcare experience of those with sensory loss may be particularly affected. Facilitating telecommunication with companions may be important to provide high quality patient-centered care, and current talks about long-term changes to the delivery of care should include solutions for those who may be relying on their companions to have access to care or communicate with their providers.

The findings from this study have limitations. The results are limited in generalizability to only Medicare beneficiaries. Moreover, the cross-sectional nature of the data do not allow for the exploration of temporality. The MCBS questionnaire design is also limited. On the MCBS, there are no questions to understand neither the actions of companions nor the immediate implications of accompaniment. Thus, those measures are beyond the scope of this study. The survey’s phrasing of “usually accompanied to your medical visit” may have been interpreted to include companions regardless of whether they join the medical encounter or instead remain in vehicles or waiting rooms. Moreover, the MCBS measures sensory impairment by self-report which may have resulted in an underestimation of the number of individuals with hearing and/or vision impairment. Thus, these findings are limited by the interpretation of the MCBS questions by respondents. Lastly, these findings are limited to routine medical encounters among older adults who report having a usual source of care and do not include those without usual sources of care who may be a particularly vulnerable population.

Further well-designed studies are needed to determine whether companions of those with sensory impairment are playing an active role during patient-provider encounters, and whether accompaniment by family, friends, caregiver modifies previously described associations such as poorer patient satisfaction and higher readmission rates. Moreover, qualitative research is needed to understand the perceptions of navigating, accessing and communicating in the healthcare setting among people with sensory impairments, and to determine more broadly how patients with dual sensory impairment are uniquely interacting with the healthcare system.

## Conclusions

In summary, this study demonstrates that vision and dual sensory impairment are associated with greater odds of accompaniment to medical visits in a nationally representative sample of older adults. Given that accompaniment among older adults with sensory impairment is highly prevalent, these findings may have clinical significance for how physicians and other healthcare providers might engage, involve and communicate with patient companions. These findings also suggest that this accompaniment may be related to communication for those with hearing impairment and transportation for those with vision impairment. While the function and value of healthcare accompaniment for those with sensory impairments may differ, further work is needed to elucidate the roles, benefits, and differences of companions of older adults with sensory impairments. From a healthcare quality planning perspective, engaging companions in the medical visit may not only improve patient-provider communication, but may also improve compliance, adherence and healthcare outcomes. At a time when the COVID-19 pandemic is changing people’s interaction with the healthcare system, it is imperative to take into account the accompaniment needs of people with sensory impairment and other groups who may be relying on their companions during medical visits.

## Data Availability

The datasets used and analyzed during the current study are openly available to the public from the United States Centers for Medicare and Medicaid Services. https://www.cms.gov/Research-Statistics-Data-and-Systems/Downloadable-Public-Use-Files/MCBS-Public-Use-File

## Abbreviations

ADL: Activities of Daily Living
CI: Confidence Interval
COVID-19: Coronavirus disease 2019
MCBS: Medicare Current Beneficiary Survey
OR: Odds Ratio
QMB: Qualified Medicare Beneficiary
